# A medium composition containing normal resting glucose that supports differentiation of primary human airway cells

**DOI:** 10.1038/s41598-022-05446-x

**Published:** 2022-01-27

**Authors:** Rachel Morgan, Candela Manfredi, Kristen F. Easley, Lionel D. Watkins, William R. Hunt, Steven L. Goudy, Eric J. Sorscher, Michael Koval, Samuel A. Molina

**Affiliations:** 1 Center for Cystic Fibrosis and Airways Disease Research, Emory University School of Medicine, Atlanta, GA 30322 USA; 2 Division of Pulmonary, Allergy, Critical Care and Sleep Medicine, Department of Medicine, Emory University School of Medicine, 205 Whitehead Building, 615 Michael Street, Atlanta, GA 30322 USA; 3 Division of Pulmonary, Allergy & Immunology, Cystic Fibrosis, and Sleep, Department of Pediatrics, Emory University School of Medicine, Atlanta, GA 30322 USA; 4 Department of Cell Biology, Emory University School of Medicine, Atlanta, GA 30322 USA

**Keywords:** Cell culture, Cell biology, Mechanisms of disease, Cystic fibrosis

## Abstract

Primary cells isolated from the human respiratory tract are the state-of-the-art for in vitro airway epithelial cell research. Airway cell isolates require media that support expansion of cells in a basal state to maintain the capacity for differentiation as well as proper cellular function. By contrast, airway cell differentiation at an air–liquid interface (ALI) requires a distinct medium formulation that typically contains high levels of glucose. Here, we expanded and differentiated human basal cells isolated from the nasal and conducting airway to a mature mucociliary epithelial cell layer at ALI using a medium formulation containing normal resting glucose levels. Of note, bronchial epithelial cells expanded and differentiated in normal resting glucose medium showed insulin-stimulated glucose uptake which was inhibited by high glucose concentrations. Normal glucose containing ALI also enabled differentiation of nasal and tracheal cells that showed comparable electrophysiological profiles when assessed for cystic fibrosis transmembrane conductance regulator (CFTR) function and that remained responsive for up to 7 weeks in culture. These data demonstrate that normal glucose containing medium supports differentiation of primary nasal and lung epithelial cells at ALI, is well suited for metabolic studies, and avoids pitfalls associated with exposure to high glucose.

## Introduction

When isolated, human airway epithelial cells are a mixture reflecting their site of origin, including the nasal, conducting and terminal airway^1,2^. Nasal and conducting airways show many similarities in morphology and cell type including non-ciliated, ciliated, secretory and multipotent basal progenitor cells. A full complement of mature, location-appropriate and niche appropriate cell types are required to accurately study the airway within a native physiological context. Each cell type contributes to the overall physiology particular to their location in the airway^3^. Basal cells are largely responsible for the maintenance of a differentiated epithelium, acting as the common airway progenitor cell^4^. Ciliated cells move mucus unidirectionally out of the airways^5^. Goblet cells and cells originating from the epithelial lined ducts of submucosal glands secrete heavily glycosylated, mucus-forming proteins^6,7^. Solitary chemosensory cells sense xenobiotics and other stimuli that induce calcium-mediated intercellular signaling to nearby cells^8^. Single cell RNAseq analysis has revealed heterogeneity among different cell types and rare subpopulations, such as ionocytes that express high levels of CFTR^9–11^. Each cell type is influenced by environmental niche factors which contribute to collective cellular homeostasis and disease dynamics in tissues^12,13^.

Cultured human primary cells have proven to be a valuable model to study airway cell differentiation and disease states including cystic fibrosis^14–21^, asthma^22,23^, chronic obstructive pulmonary disease (COPD)^24^ and COVID-19^25–27^. To mimic the native cell environment, cell culture media contain key factors found in tissue fluids in vivo required to support proper cell differentiation^4,28,29^. Culture media resembling human plasma has supported the premise that nutrient sources in vitro can negatively impact cellular homeostasis which influences assays widely used in research such as cell-based drug screens^30^. Early passage primary cells grown in traditional epithelial growth medium often do not fully differentiate after expansion which limits their utility as a model to study native tissue physiology. This is even more of a concern when considering human cell samples obtained from non-invasive epithelial sampling techniques, such as nasal or conducting airway brushings, which are valuable for rare disease research but limited by the absolute number of cells that are collected^17,31,32^. Basal cells derived from human induced pluripotent stem cells (iPSCs) represent another potential source of differentiable cells^33^. Methods to expand small samples into a large bank of cells with the capacity to be well-differentiated allows better access to primary respiratory epithelial cells for human disease research.

Expanding primary cells while maintaining their capacity to differentiate requires methods that rely on chemical inhibition or stalling of differentiation while still enabling basal progenitor cells to propagate. In addition to retaining multipotency in the expanding pool of basal progenitor cells, expansion methods prolong the differentiation potential of the resulting expanded cells. The cells are then able to undergo more population doublings than using traditional expansion methods, while retaining their ability to differentiate into a properly differentiated cell layer in vitro.

Chief among these is the conditional reprogramming culture (CRC) method where primary epithelial cells are co-cultured with irradiated (non-proliferating) fibroblasts in medium containing the rho-associated protein kinase (ROCK) inhibitor Y-27632^28,34,35^. These conditions preserve the basal cell phenotype, prevent differentiation and are fully reversible^36,37^. Epithelial cells expanded using CRC conditions retain their ability to differentiate for at least twice as long as compared to cells grown in traditional epithelial growth medium^36^.

Another approach, the Dual SMAD inhibitor method, has also proven effective and involves culturing basal cells in the presence of inhibitors that target TGFβ (usually SB431542) and BMP4 signaling (dorsomorphin, LDN193189 or recombinant Noggin)^29,38,39^. Note that the CRC and Dual SMAD basal cell expansion methods are not completely equivalent. For instance, recent evidence suggests that the CRC method better preserves some aspects of airway cell differentiation potential including ciliation and CFTR channel function^40^.

Traditional culture techniques rely on expansion media with formulations that are rich in sugars, serum proteins, and supplements that exceed levels found in healthy human serum. This results in medium that fosters cell viability but causes cell overgrowth due to an overreliance in anerobic glycolytic energy metabolism over aerobic oxidative phosphorylation^41,42^. Given accumulating evidence that the metabolic microenvironment can have a significant impact on airway epithelial cell function^14,18,20,43–45^, it is important to consider medium composition as a variable that can influence behavior of cells in vitro^46^. Of note, several media commonly used to support airway epithelial cell differentiation have high glucose concentrations, including LHC Basal:DMEM-H 50:50^47^ and Pneumocult-ALI^48^ both of which contain ~ 300 mg/dL glucose.

Here we describe a method that can be used to propagate and differentiate basal cells from the upper and conducting airways in medium containing normal resting glucose concentrations. The ALI medium formulation described here supports the ability of airway cells to respond to insulin by stimulating glucose uptake, suggesting that these cultures are well suited for use in studying the impact of energy metabolism on airway cell function.

## Results and discussion

### Isolation and differentiation of airway epithelial cells

**Figure 1:**
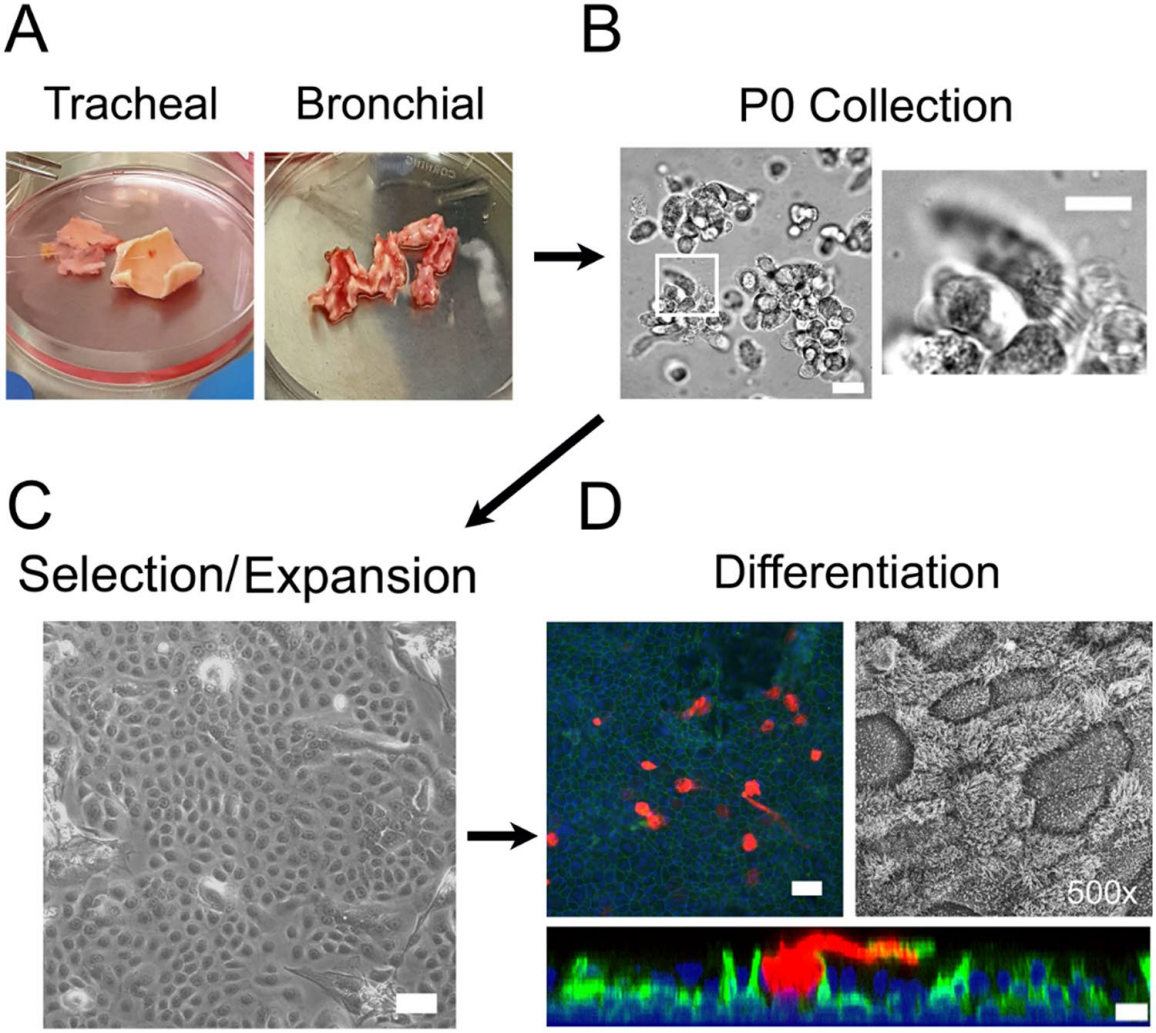
Workflow for isolation of cells from distinct anatomic regions of the airway tree. (**A**) Examples of healthy tracheal and bronchial tissue isolates are shown based on donor tissue color, shape, and rigidity as markers for tissue health. (**B**) Freshly isolated primary (passage 0; P0) cells isolated from nasal or lung tissue contain a mixture of cells, including ciliated (inset) and non-ciliated cells. Bars, 20 μm (left) and 10 μm (right). (**C**) Basal nasal or airway epithelial cells are selected and expanded using CRC conditions, as imaged by phase contrast microscopy. Bar, 10 μm. (**D**) Basal airway or nasal cells cultured in E-ALI containing normal glucose properly differentiate as determined by immunofluorescence confocal microscopy using markers for mucus secretion (Muc5AC, red), basal cells (KRT5, green), nuclei (blue, DAPI) and by scanning electron microscopy. Bars, 20 μm (top and middle) and 10 μm (bottom).

Figure 1 illustrates the workflow of tissue harvest, primary cell isolation, basal cell selection and propagation and differentiation to produce airway cell models to study in vitro. The overall health of the donor tissue directly affects the quality of the resulting isolated cell culture. In our experience, the number of viable epithelial cells able to be isolated is mainly affected by the condition of the tissue when received and the time since resection. Culture of airway epithelial cells consists of two phases: 1) propagation of basal epithelial cells followed by 2) preparation of differentiated cultures using an air liquid interface (ALI).

**Figure 2:**
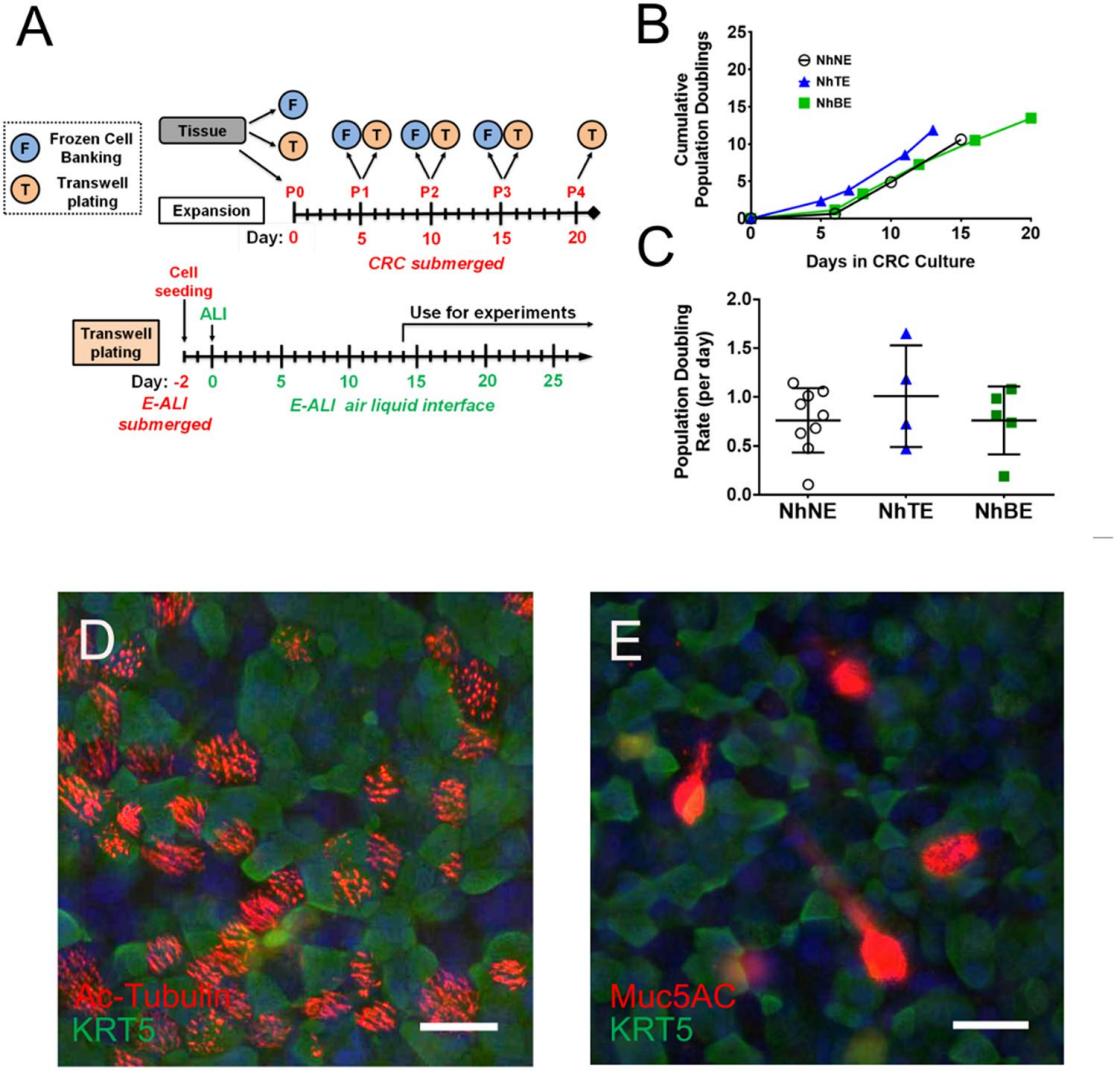
Airway epithelial cells from different tissue sources propagate with similar doubling times. (**A**) Timeline for processing of cells isolated from lung tissue samples showing frozen cell banking (F), plating for differentiation on Transwell permeable supports (T) or plating for expansion under CRC conditions. Cells from P0 through P3 are banked. Cells beyond P4 are not typically used to generate differentiated cultures for experimental analysis. Detail related to culture on Transwells (T) is shown below, indicating the shift from submerged to air–liquid interface (ALI). Cells are cultured at least 14 days at ALI prior to use in experiments. (**B**) Representative NhNE, NhTE, and NhBE displayed a lag phase of growth between Day 0 and Day 5 in CRC conditions before replicating at a linear rate. (**C**) Regardless of anatomical origin or the initial lag phase, airway epithelial cells showed comparable doubling rates under CRC conditions. n = 2 – 3 wells from n = 2 (NhTE, NhBE) or 4 (NhNE) biological replicates plotted as mean ± SD. (**D**, **E**) Immunofluorescence analysis of differentiated tracheal epithelial cells (NhTE) at Day 14 in E-ALI. Ciliated cells were identified by immunostaining for acetylated tubulin (Ac-Tubulin, red) and basal cells by cytokeratin 5 (KRT5, green). Nuclei were labeled with DAPI (blue). (**D**). Mucus producing cells were identified by Muc5AC expression (red) (**E**). Bar, 20 μm.

Initially, P0 cells were cultured using the CRC method which is submersion culture in medium based on a mixture of DMEM and F12, resulting in normal resting glucose levels (150 mg/dL; 8.3 mM), and including biologic co-factors, the ROCK inhibitor Y-27638 and cholera toxin (FYRM; Table 1). The cells are seeded on collagen coated dishes and co-cultured with 3T3 fibroblasts that were irradiated to inhibit their propagation (Fig. 2A). When ~ 40% or more P0 cells attached to collagen coated dishes seeded with irradiated 3T3 cells, this was an indication that tissue processing was successful. Generally, cells exhibited a 5-day lag period before beginning to proliferate (Fig. 2B). Following the lag period, growth rates of cells isolated from anatomically different areas of the airway were similar (Fig. 2B) with a population doubling rate of roughly one per day (Fig. 2C).

**Table 1:** FYRM medium composition.

	Reagent	Final Conc. (For 1000 mL)	Supplier	Catalog #
Base Medium	DMEM 1.0 g/L glucose (w/L-glutamine and w/ sodium pyruvate)	225 mL	Corning	10–014-CV
	Ham’s F12	725 mL	Cytiva	SH30026.01
Biologics	Fetal Bovine Serum	50 mL	R&D Systems	S11150
	Insulin	5 µg/mL	Gemini Bio	800-112P
	Epidermal Growth Factor	10 ng/mL	StemCell Tech	78006.2
	Hydrocortisone	480 ng/mL	StemCell Tech	74142
	Adenine	24 µg/mL	Sigma-Aldrich	A2786
Agents	Y-27632	10 µM	Tocris	1254
	Cholera Toxin	8.33 ng/mL	Sigma-Aldrich	C8052
Anti – infectives	Primocin	0.2% (2 mL)	Invivogen	ant-pm-1
	Plasmocin	0.1% (1 mL)	Invivogen	ant-mpp
	Vorconazole	200 ng/mL	Selleck Chemicals	S1442

This formulation produces medium containing 150 mg/dL glucose as measured using a colorimetric assay. For details related to medium preparation, see Methods.

Once the cells were isolated and propagated, frozen cell stocks were made at each passage creating a bank of cells with consistent properties that can be used for experimentation (Fig. 2A). Generally, we avoid cryobanking after P3 to ensure that the basal cells maintain their capacity to differentiate. For freshly isolated P0 cells, freeze densities of two million cells per vial allow for at least 500,000 cells per thaw to attach. For P1-P3, freeze densities of one million cells per vial are recommended to facilitate rapid growth from banked vials.

Culture of airway basal cells at ALI is a well-established method to promote their differentiation, however most media used for this purpose contain high glucose concentrations (~ 300 mg/dL; 16.7 mM)^47,48^, which reflects a hyperglycemic state^14,44^. Given this, we developed a modified ALI medium, E-ALI, based on a widely used medium formulation^47^. As shown in Table 2, E-ALI contains normal resting glucose levels (150 mg/dL; 8.3 mM). Otherwise, E-ALI is comparable to other ALI medium formulations^47,48^, except that it has less insulin (5 µg/ml) and is enriched for the following components: CaCl_2_ (1 mM), heparin (2 µg/ml), L-glutamine (2.5 mM), hydrocortisone 960 mg/ml, O-phosphorylethanolamine (0.5 µg/ml), bovine pituitary extract (20 µg/ml) and Mg^2+^ (0.5 µM).

**Table 2:** E-ALI medium composition.

	Reagent		Final Conc. (For 1000 mL)	Supplier	Catalog #
Base Medium	DMEM 1.0 g/L glucose (w/o L-glutamine and w/sodium pyruvate)		50% (500 mL)	Sigma-Aldrich	D5546
	LHC Basal Medium		50% (500 mL)	ThermoFisher	12677019
Biologics	L-glutamine		2.5 mM (12.5 mL)	Corning	25-005-CV
	Insulin		5 µg/mL	Gemini Bio	800-112P
	Epidermal Growth Factor		0.5 ng/mL	StemCell Tech	78006.2
	Hydrocortisone		960 ng/mL	StemCell Tech	74142
	Bovine Pituitary Extract		20 µg/mL	Gemini Bio	500-102
	Triiodothyronine (T3)		10 nM	Sigma-Aldrich	T6397
	Transferrin		125 nM	Gemini Bio	800-131P
	Epinephrine		2.7 µM	Sigma-Aldrich	E4250
	O-phosphorylethanolamine		0.5 µM	Sigma-Aldrich	P0503
	Ethanolamine		0.5 µM	Sigma-Aldrich	E0135
	Retanoic Acid		50 nM	StemCell Tech	72262
	Heparin		2 µg/mL	StemCell Tech	07980
	Bovine Serum Albumin, Fraction V		500 µg/mL	Gemini Bio	700-102P
CaCl_2_ stock	Calcium Chloride dihydrate		1 mM	Sigma-Aldrich	C3881
ZnSO_4_ stock	Zinc Sulfate heptahydrate		3 µM	Sigma-Aldrich	Z0251
Mg/Fe stock	Ferrous Sulfate heptahydrate		1.51 µM	Sigma-Aldrich	F8633
	Magnesium Chloride hexahydrate		300 µM	Sigma-Aldrich	M2393
	Magnesium Sulfate heptahydrate		195 µM	Sigma-Aldrich	M5921
	Hydrochloric Acid (12 M)		60 µM	ThermoFisher	A144-500
Trace Elements	Sodium Selenite		30 nM	Sigma-Aldrich	S5261
	Manganese(II) chloride tetrahydrate		1 nM	Sigma-Aldrich	M5005
	Sodium metasilicate nonahydrate		500 nM	Sigma-Aldrich	S5904
	Ammonium molybdate tetrahydrate		1 nM	Sigma-Aldrich	M1019
	Ammonium metavanadate		5 nM	Sigma-Aldrich	398128
	Nickel(II) sulfate hexahydrate		1 nM	Sigma-Aldrich	N4882
	Tin(II) chloride dihydrate		0.5 nM	Sigma-Aldrich	S9262
	Hydrochloric Acid (12 M)		12 µM	ThermoFisher	A144-500
Anti-infectives (add A,B,C or D as needed)	Primocin		0.2% (2 mL)	Invivogen	ant-pm-1
	Plasmocin		0.1% (1 mL)	Invivogen	ant-mpp
	A	Ceftazidime	7.7 µg/mL	ThermoFisher	AC461730050
		Gentamicin	5 µg/mL	Sigma-Aldrich	G1397
	B	Cilistatin/Imipenem	25 µg/mL	Astatech	42454
	C	Piperacillin	4.4 µg/mL	Alfa Aesar	J66143-ME
		Tazobactam	0.6 µg/mL	Alfa Aesar	J66226-03
	D	Azithromycin	5 µg/mL	Alfa Aesar	J66740-06
		Voriconazole	0.5 µg/mL	Selleck Chemicals	S1442

This formulation produces medium containing 150 mg/dL glucose as measured using a colorimetric assay. For details related to medium preparation, see Methods.

We validated the ability of E-ALI to support the growth and differentiation of freshly isolated P0 normal human tracheal epithelial cells (NhTE cells) plated on collagen-coated Transwell permeable supports as assessed by immunofluorescence microscopy using KRT5 as a marker for basal cells (Fig. 2D-E), acetylated-tubulin as a marker for ciliated epithelia (Fig. 2D), and Muc5AC as a marker for mucus producing cells (Fig. 2E). Cultures routinely contained all three different cell types, indicating that they were well differentiated.

**Figure 3:**
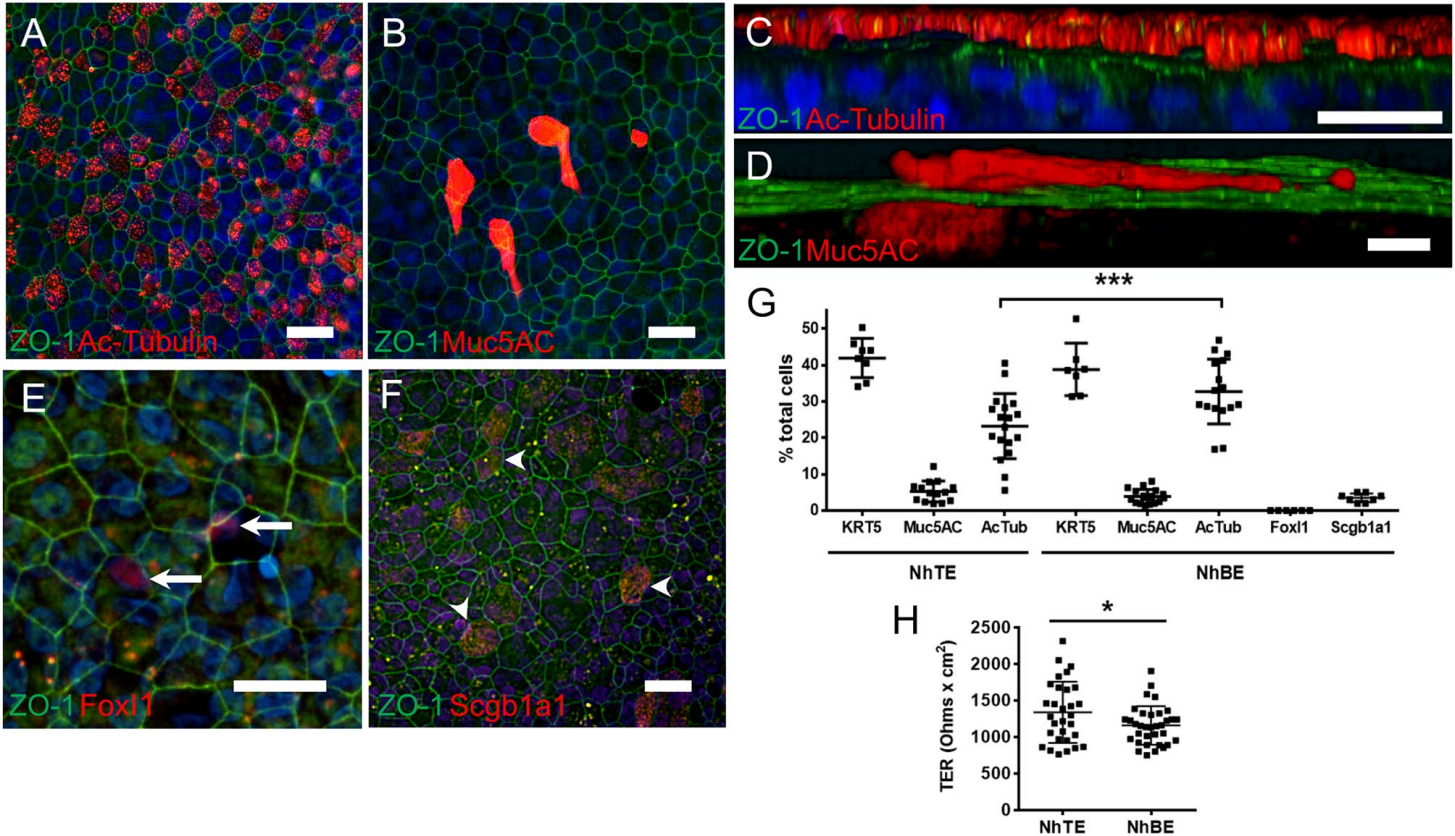
Tracheal and bronchial epithelial cells differentiate into mucociliary cultures in E-ALI medium. (**A**–**E**) The E-ALI formulation enabled formation of well-differentiated cultured bronchial epithelial cell (NhBE) monolayers in vitro as observed by the presence of tight junctions (green, zonula occludins-1, ZO-1). Cell differentiation was demonstrated by immunofluorescence microscopy measuring acetylated tubulin (red, Ac-Tubulin; **A**, **C**), mucin (red, Muc5AC; **B**, **D**), an ionocyte marker (red, FoxI1; **E**) and a club cell marker (red, Scgb1a1; F). Nuclei were labeled with DAPI (blue). Bar, 20 μm. (**G**) Quantitation of NhTE and NhBE phenotype in cells cultured for 14 days in E-ALI. Data are from n = 2 – 9 fields from n = 2 (KRT5, FoxI1, Scgb1a) or 3 (Muc5AC, AcTub) biological replicates. ***P < 0.0001 by one-way ANOVA. (**H**) Transepithelial electrical resistance (TER) of NhTE cells was slightly higher than NhBE cells n = 3—21 wells from 4 biological replicates; *P = 0.042, by t test.

Comparable results were obtained using normal human bronchial epithelial cells (NhBE cells), which are delineated by apical junctions as marked by the tight junction protein ZO-1 (Fig. 3A-F) and also show ciliated cells (Fig. 3A,C), mucus producing cells (Fig. 3B,D), ionocytes (Fig. 3E) and club cells (Fig. 3F). After 14 days of culture in E-ALI, NhBE monolayers had significantly more ciliated cells than NhTE monolayers (32.7 ± 8.9%, n = 3 replicates, 14 fields; 23.2 ± 9.0%, n = 3 replicates, 18 fields) (Fig. 3G). The number of basal cells were comparable for NhBE and NhTE monolayers (38.8 ± 7.2%, n = 2 replicates, 7 fields; 41.9 ± 5.4%, n = 2 replicates, 8 fields). Muc5AC positive cells were also comparable for NhBE and NhTE monolayers (3.9 ± 1.9%, n = 3 replicates, 18 fields; 5.3 ± 2.9%, n = 3 replicates, 14 fields). NhBE monolayers also contained low levels of club cells (2.0 ± 1.1%, n = 2 replicates, 12 fields) and ionocytes (0.1 ± 0.1%, n = 2 replicates, 13 fields). Altogether, we accounted for 77.5 ± 11.7% of the total cells in NhBE monolayers grown using E-ALI. Other cell types likely to be present include suprabasal cells which express KRT4, KRT8 and KRT13^49,50^, however, suprabasal and other cell populations defined by multiple markers are difficult to detect strictly by immunofluorescence profiling.

Consistent with formation of tight junctions, NhBE and NhTE cells cultured in E-ALI showed high transepithelial resistance (TER) (Fig. 3H), where the barrier formed by NhTE cells after 14 days in E-ALI was slightly, but significantly tighter than NhBE cells (~ 1340 vs ~ 1160 Ohm x cm^2^). Taken together, these data indicate that the E-ALI formulation containing normal resting glucose supported airway epithelial cell function as determined by apical junction assembly and differentiation of tracheal and bronchial epithelial cells.

### Cells cultured in E-ALI medium show insulin-stimulated glucose uptake

**Figure 4:**
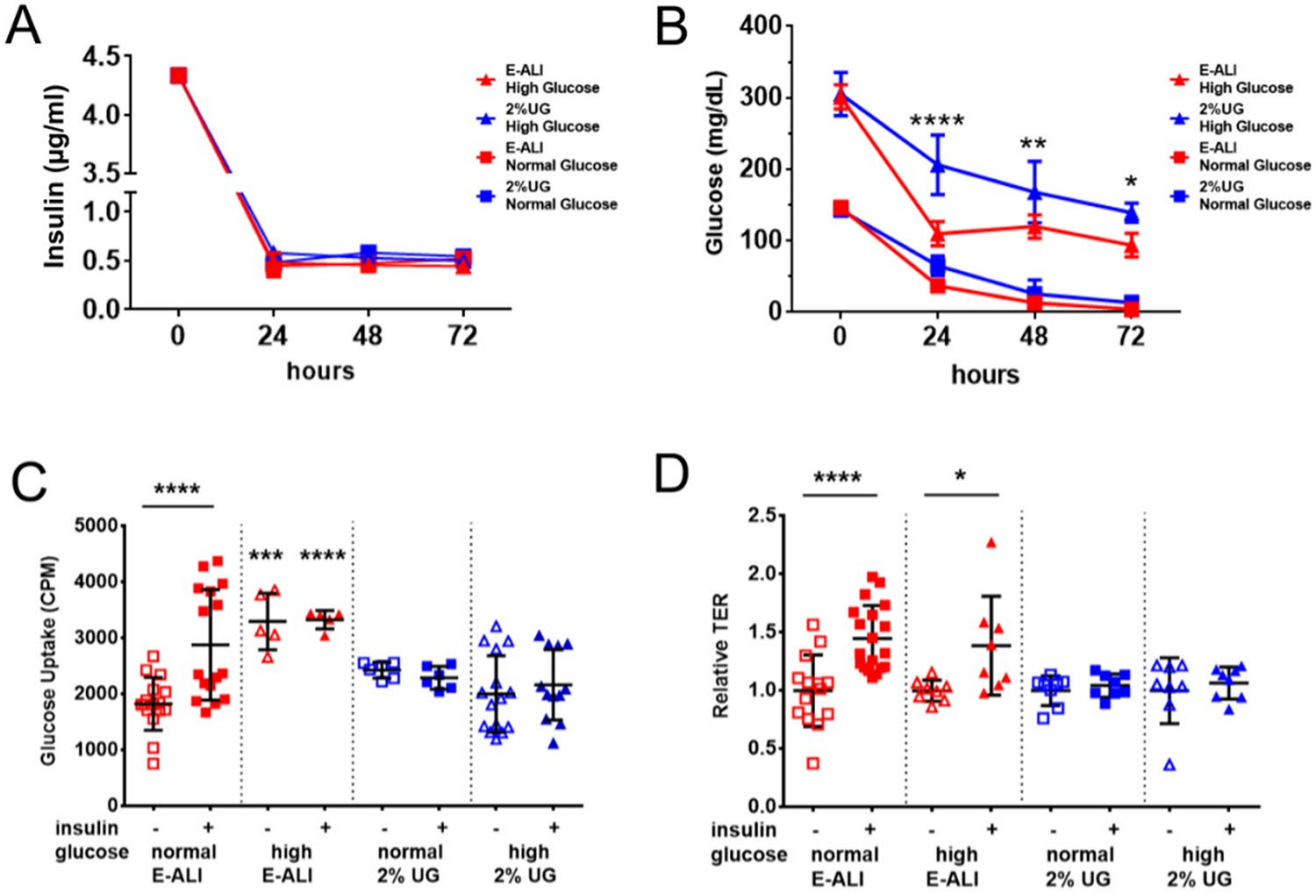
Glucose and insulin sensitivity depends on differentiation medium. (**A**) Insulin consumption was comparable in all media tested. n = 4 samples, one biological replicate per condition. (**B**) Glucose consumption of differentiated, expanded P0 airway epithelial cells of mixed tracheal/bronchial origin. Red triangles and squares indicate cells cultured in E-ALI medium and blue triangles and squares indicate cells cultured in 2% Ultroser G (2% UG) medium. High glucose = 300 mg/dL glucose in the base media (triangles); Normal resting glucose  = 150 mg/dL glucose in the base media (squares). Glucose consumption was significantly higher for cells in high glucose E-ALI as compared with cells in high glucose 2% UG (n = 4 samples, one biological replicate; ****P < 0.0001; **P = 0.0085; *P = 0.014, by two way ANOVA). (**C**) Cells differentiated using E-ALI medium containing normal resting glucose elicited a significant increase in insulin stimulated [^3^H]-2-D-glucose uptake. n = 3–6 samples from n = 1 (E-ALI high glucose, 2% UG normal glucose), 3 (2% UG High glucose) or 4 (E-ALI normal glucose) biological replicates; ***P = 0.0001; ****P < 0.0001, by one-way ANOVA. (**D**) Cells cultured in E-ALI showed significant increases in relative transepithelial resistance (TER) in response to insulin regardless of glucose content, cells cultured in 2% UG did not. n = 4—11 samples from n = 3 (E-ALI normal glucose) or 2 (all others) biological replicates; *P = 0.015, ****P < 0.0001 by one-way ANOVA. All data is plotted as mean ± SD.

To measure the effect of extracellular glucose and medium composition on insulin and glucose clearance, we examined NhBE cells cultured in either E-ALI or 2% Ultroser G containing normal (150 mg/dL) or high (300 mg/dL) glucose (Fig. 4). It is worth noting that E-ALI containing 300 mg/dL glucose is comparable to the glucose content of LHC Basal:DMEM-H 50:50^47^ and Pneumocult-ALI^48^. Insulin clearance was not sensitive to medium formulation, where all of the cell culture models tested cleared the majority of insulin within the first 24 h after feeding, causing it to plateau at a low level (~ 0.4 µg/mL; Fig. 4A).

Medium glucose content showed a fast decline within the first 24 h where each culture grown used approximately 50% of the available glucose (Fig. 4B). Glucose clearance was significantly more rapid for cells in high glucose E-ALI as compared with cells in high glucose 2% Ultroser G medium. After 72 h, nearly 125 mg/dL glucose remained in cultures fed with high glucose media, consistent with saturation of uptake. By contrast, cells in normal resting glucose cleared nearly all glucose from the medium after 72 h.

We then determined the impact of medium formulation and glucose content on insulin stimulated glucose uptake, as measured using [^3^H]-2-deoxy-glucose. Since E-ALI used to culture airway cells contains insulin (5 µg/ml; 0.87 µM), cells were first pre-incubated for 90 min with insulin-free KRH prior to challenge with [^3^H]-2-deoxy-glucose in the presence or absence of 500 nM (2.9 µg/ml) recombinant human insulin. Of all the conditions tested, only E-ALI medium containing normal resting glucose showed a significant, two-fold increase in glucose uptake in response to added insulin (Fig. 4C). By contrast, cells cultured E-ALI containing high glucose showed elevated glucose uptake that was insulin insensitive and significantly higher than the levels of glucose uptake by cells in E-ALI at normal resting glucose in the absence of insulin. This was not due to an effect of high glucose on insulin signaling, since cells cultured in E-ALI showed an increase in transepithelial resistance (TER) in response to insulin, regardless of glucose concentration and consistent with our previous results^20^.

Airway cells express multiple glucose transporters, including the insulin regulated Glut4 transporter^20^. Our data suggest that cells chronically cultured in the presence of high glucose are likely to upregulate constitutive glucose transporters, which would overshadow the impact of insulin stimulated activation of Glut4 mediated by trafficking from secretory vesicles to the plasma membrane^20,44^. Regardless of mechanism, it is important to note that the glucose concentrations used here represent two extremes as opposed to the physiological glucose levels cells will be exposed to in vivo which significantly vary in response to meals and systemic insulin levels^51^. Our data suggest that a culture system based on exposing cells to E-ALI containing varying levels of glucose could provide the basis for an in vitro model that mimics in vivo exposure.

Moreover, cells cultured in 2% Ultroser G did not show insulin stimulated glucose uptake or changes in TER (Fig. 4C,D). Considering that constitutive insulin uptake was comparable for cells cultured in E-ALI and 2% Ultroser G, the differences in insulin stimulated glucose uptake and barrier function were not likely to be due to a difference in insulin binding capacity. Instead, the results in Fig. 4C,D and more likely reflect a difference in the capacity for glucose uptake (Fig. 4B) and/or signaling downstream from insulin receptors. For instance, we have observed that the ability of insulin to promote barrier function requires akt signaling, a pathway that is active in primary human airway cells ^20^. Taken together, these results underscore the importance of medium formulation, especially in studies of airway cell metabolism, and are consistent with the deleterious effects of hyperglycemia on the airway epithelium^14,44,52^ as well as cell homeostasis in general^53^.

### Expansion and maturation of CF nasal epithelial cells using E-ALI

**Figure 5:**
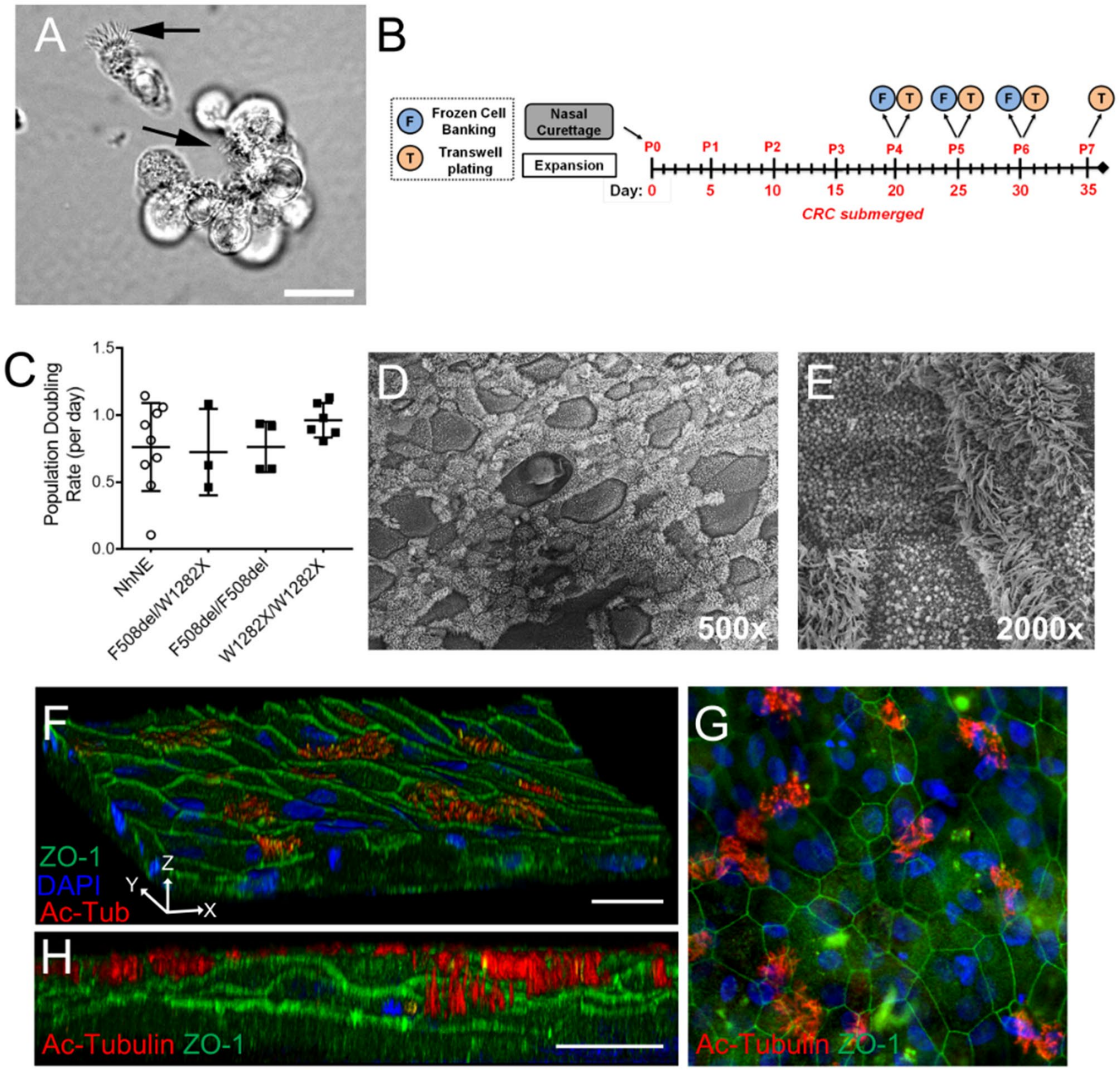
Expansion and differentiation of non-CF and CF nasal epithelial cells. (**A**) Ciliated cells (black arrows) are observed in freshly isolated CF nasal cultures by phase contrast microscopy. Bar, 20 μm. (**B**) Timeline for processing of nasal curettage samples showing frozen cell banking (F), plating for differentiation on Transwell permeable supports (T) or plating for expansion in FYRM submerged culture. Due to the small initial sample size, cells were not banked or plated for differentiation until P4. Cells beyond P7 are not typically used to generate differentiated cultures for experimental analysis. Detail related to culture on Transwells (T) is shown in Fig. 2. (**C**) Non-CF and CF nasal epithelial cells with 3 different genotypes had a comparable doubling time when cultured in CRC conditions. n = 3 – 6 wells for CF cells; doubling data for NhNE cells is from Fig. 2. (**D**, **E**) Cilia and mucus producing CF nasal epithelial cells as observed by scanning electron microscopy at 500x (**D**) and 2000x (**E**) magnification. (**F**–**H**) Immunofluorescence confocal microscopy of CF nasal airway cells showed tight junctions (green, ZO-1) and cilia (red, Ac-Tubulin). Nuclei were labeled with DAPI (blue). Bar, 20 μm (**F**, **H**).

Nasal cells have proven to be a useful model system that reflects several characteristics of the conducting airway^16,54^. Unlike primary tracheal and bronchial cells, primary human nasal epithelial (hNE) cells often originate from small samples that require expansion on an appreciable scale for subsequent analysis. We thus evaluated the ability of E-ALI to support differentiation of hNE cells including CFhNE cells harboring both rare and common disease-causing CFTR alleles, as well as cells from non-CF subjects (NhNE cells). Nasal cell isolates (Fig. 5A) initially were expanded using CRC conditions (Fig. 5B) and then differentiated using the protocol illustrated in Fig. 2A. Regardless of genotype, hNE cells showed comparable doubling rates of ~ 0.7–0.9 per day during CRC expansion (Fig. 5C). Ciliated cells were readily detected 21 days after initiating culture in E-ALI by scanning EM (Fig. 5D,E) and by confocal immunofluorescence microscopy (Fig. 5F-H). Taken together these data show that expansion of nasal epithelial cells using the CRC method effectively supported their ability to differentiate in E-ALI.

**Figure 6:**
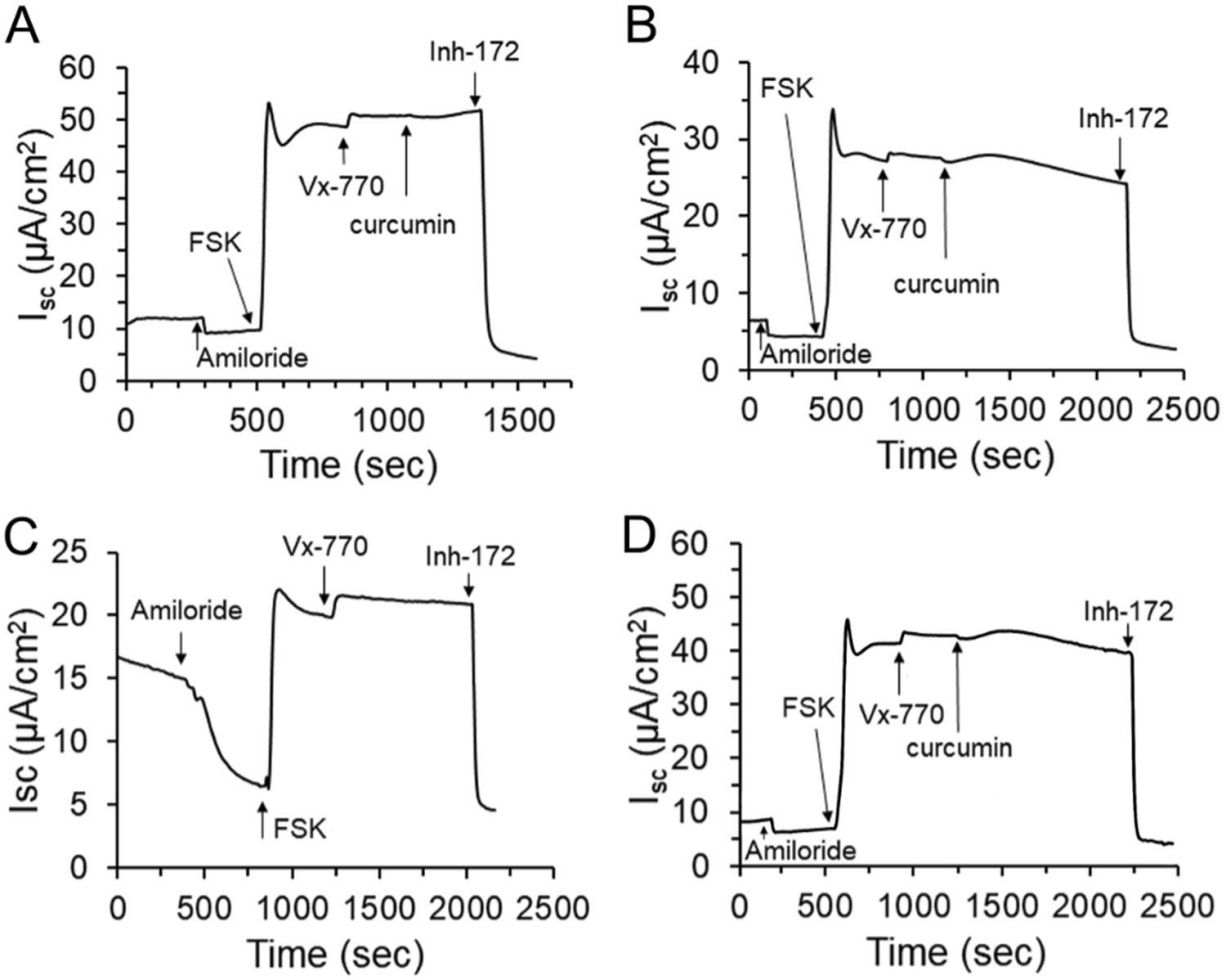
Representative electrophysiological analysis of primary tracheal and nasal airway epithelia expanded using CRC conditions and differentiated in E-ALI. (**A**, **B**) P0 NhTEC cultured in E-ALI for 16 weeks (**A**) or 21 weeks (**B**) show comparable response profiles to the ENaC inhibitor amiloride and agents that stimulate or inhibit CFTR currents. (**C**, **D**) NhNE cells that were expanded using CRC conditions to P4 and then differentiated with E-ALI for 2 weeks (**C**) or 7 weeks (**D**), also exhibited comparable ENaC and CFTR current profiles. FSK, Forskolin; Vx-770, Ivacaftor; CFTRinh172, CFTR channel inhibitor.

**Figure 7:**
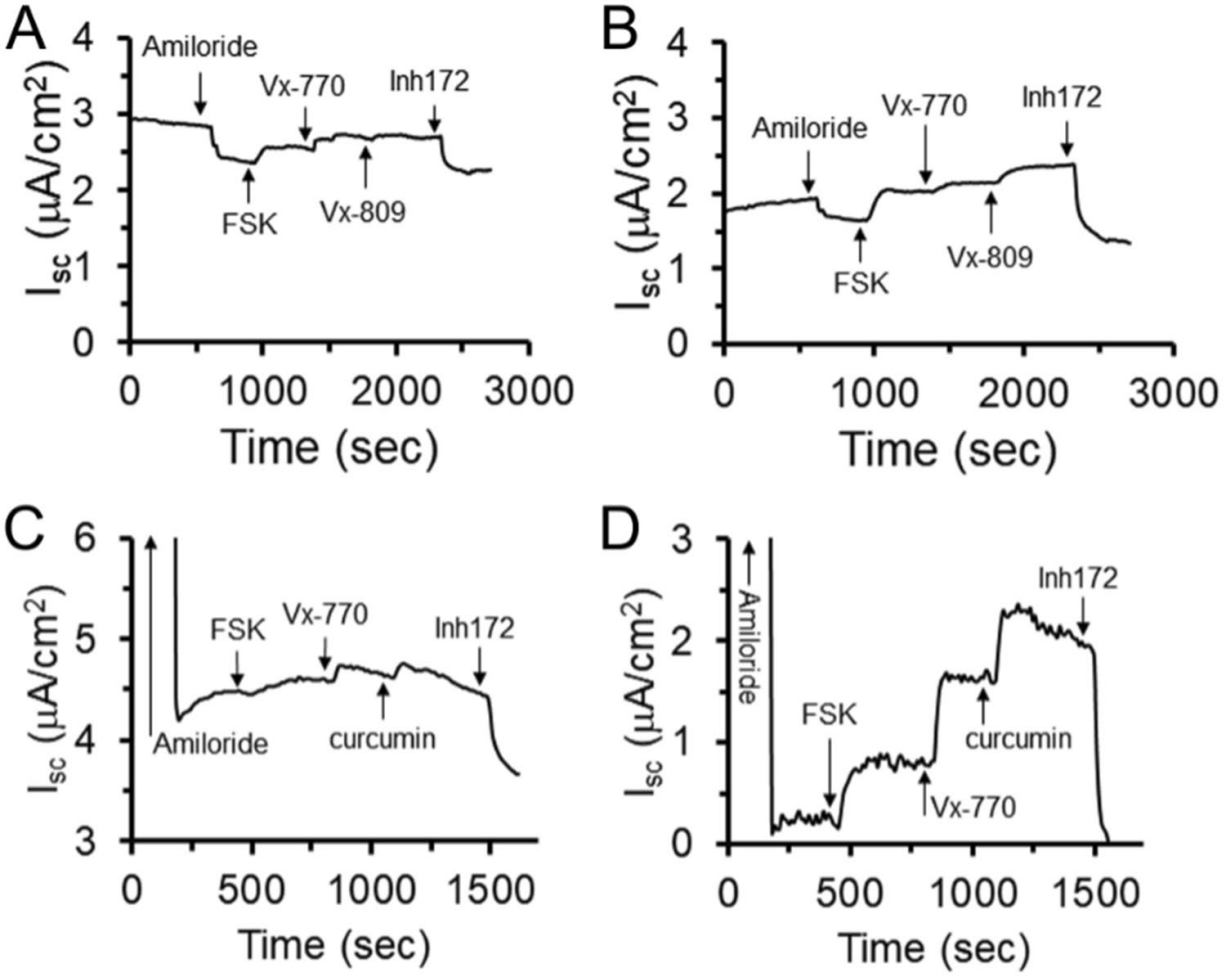
Representative electrophysiological analysis of primary nasal CF airway epithelia expanded using CRC conditions and differentiated in E-ALI. (**A**, **B**) CFhNE cells with the G551D/F508del genotype were expanded using CRC conditions to P4 and differentiated in E-ALI for 14 days. Shown are two representative traces demonstrating low levels of CFTR currents and modest responses to Vx-770 and Vx-809. (**C**, **D**) CFhNE cells with the W1282X/F508del genotype were expanded and differentiated as described above. Shown are two representative traces demonstrating low levels of CFTR currents and responses to Vx-770 and curcumin. FSK, Forskolin; inh172, CFTR channel inhibitor; Vx-770, Ivacaftor; Vx-809, Lumacaftor.

We further characterized the cell electrophysiology of P0 NhTE cells maintained in E-ALI medium. After 16 weeks at ALI (Fig. 6A) NhTE cells demonstrated measurable ENaC and CFTR currents based on amiloride inhibition and forskolin stimulation, respectively. CFTR currents were also modestly enhanced by Vx-770 (ivacaftor) and curcumin and were inhibited by Inh-172. NhTE cells from the same culture preparation extended to 21 weeks ALI had a comparable electrophysiological profile (Fig. 6B). Primary NhNE cells expanded with the CRC method, then differentiated with E-ALI for either 2 weeks (Fig. 6C) or 7 weeks (Fig. 6D) also showed ENaC and CFTR currents with electrophysiological characteristics comparable to those of NhTE cells. These data demonstrate the utility of E-ALI in supporting long-term cultures that maintained ion channel function.

We then examined primary CFhNE cells with a G551D/F508del genotype that were isolated, expanded, and differentiated in E-ALI. Differentiated G551D/F508del CFhNE cells produced mature cells capable of eliciting small, but detectable, CFTR currents when treated with forskolin that were modestly enhanced with Vx-770 and Vx-809 (lumacaftor) and inhibited with Inh-172 (Figs. 7A-B) ^55,56^. Similarly, CFhNE cells with a W1282X/F508del genotype exhibited a small CFTR current in response to forskolin and Vx-770 and were also responsive to curcumin which has shown efficacy in CFTR mutants encoding a premature stop codon (Figs. 7C-D)^57^. Therefore, E-ALI medium is compatible with CRC expansion and subsequent studying of primary nasal epithelial cells.

Taken together, these data validate E-ALI as a method to differentiate human airway epithelial cells in medium containing normal resting glucose levels. We also confirmed that expanded nasal epithelial cell isolates have preserved their ability to differentiate and express functional CFTR. E-ALI medium provides a new method amenable to investigation of nasal, tracheal, and bronchial airway epithelia for a variety of applications including ciliation and developmental studies, host pathogen interactions, and drug screening. The ability to differentiate cells in normal resting glucose is expected to facilitate the analysis of airway cell functions that are particularly sensitive to cell metabolism.

## Methods

### Donor consent

Research involving human research participants was performed in accordance with the Declaration of Helsinki guidelines and samples were de-identified to meet HIPAA requirements. Nasal curettage, tracheal, bronchial, and whole lung tissues were acquired through informed consent via an Emory University IRB approved protocol (protocol #00005792) administered by the Cystic Fibrosis Biospecimen Repository (CFBR). Additional lung tissues were obtained through standardized UNOS consenting procedures for tissue donation for research in conjunction with an IRB-approved waiver from Emory University.

### Tracheal epithelial cell isolation

To prepare tracheal epithelial cells, intact human donor tracheal tissue was cut into segments consisting of two to three cartilage rings starting at the carina of the main tracheal bifurcation proceeding distally. The trachealis muscle was removed to simplify the isolation procedure as the muscle tends to disintegrate during enzymatic digestion and increases co-purifying tissue debris. All tracheal segments were placed into a 250 mL sterile plastic bottle and washed in Hanks Balanced Salt Solution (HBSS; Sigma-Aldrich #55021C-1000ML) at least 5 times. Then, the convex outer side of the tracheal segments were cleaned using tweezers and a scalpel to remove excess connective tissue and prevent accumulation of tissue debris.

Epithelial cells were removed from the underlying extracellular matrix by first incubating at 4 °C for least 12–16 h under gentle agitation in 50–150 mL of Conducting Airway Protease Solution (CAPS) consisting of Ham’s F-12 medium (Hyclone #SH30026.FS) supplemented with 1.0% w/v Protease XIV (Sigma-Aldrich #P5147), 0.1% w/v DNAse I (Sigma-Aldrich #DN25), 0.2% Primocin (Invivotech #amt-pm-1), and 0.1% Plasmocin treatment agent (Invivotech #ant-mpt). To loosen and remove intact epithelial cell sheets from the concave inner side of the trachea, the digestion solution containing the tracheal segments was lightly vortexed.

The cell solution was separated from the tissue segments by first decanting into a new conical tube, then scraping the concave side with a scalpel to remove any remaining epithelia. The tracheal segments were washed with HBSS and the solution collected into 50 mL centrifuge tubes. All solutions containing cells were centrifuged at 350 × g for 10 min at RT, resuspended in a total of 20 mL normal glucose DMEM (Sigma-Aldrich # D6046 or Hyclone #SH30021.FS), centrifuged again at 350 × g for 10 min at RT, then resuspended in calcium/magnesium-free PBS supplemented with 1 mM EDTA (PBS/EDTA). Cells in solution were triturated to break apart cell clumps, passed through a 100-micron filter (Corning #352360), followed by a 70-micron filter (Corning #431751). The resulting P0 cells were then either cryopreserved at 1 million cells/mL, cultured for differentiation, or cultured for expansion.

### Bronchial epithelial cell isolation

Intact human donor bronchial tissue caudal from the tracheal bifurcation to airways 10 mm in diameter were used to isolate pure bronchial epithelial cells. These tissue segments were generally lined with soft cartilage rings. Bronchial tissue segments were isolated from whole lungs or lobes of intact lungs by carefully removing the surrounding terminal airway tissue working from the tracheal bifurcation towards the caudal end of the bronchi. Isolated bronchial tissue segments were washed at least 5 times in HBSS to remove any accumulated mucus and to loosen any remaining connective tissue. Airways beyond bronchi generally include bronchioles that are not heavily collagenous and are smaller than 3 mm in diameter. These airways take time and effort when isolating to avoid cross contamination with pulmonary arteries and smaller vessels. For bronchi, tissue segments were cleaned of connective tissue, cut longitudinally to expose the epithelium, and then processed to isolate P0 cells as described for tracheal cell isolation. As needed, terminal airway lobe tissue was set aside in normal glucose DMEM for primary fibroblast isolation by established methods^58^.

### 3T3 Fibroblast feeder cell preparation

Fibroblast feeder layers required for expansion of primary human basal airway epithelial cells were prepared using the 3T3-J2 fibroblast cell line (ATCC #SCRC-1010)^4,36,59,60^. 3T3 cells were expanded in DMEM containing high glucose (450 mg/dL) (Sigma-Aldrich #D6429 or Hyclone #SH30243.01) supplemented with 10% FBS (ThermoFisher #26170043), 0.2% Primocin (Invivotech #amt-pm-1) and 0.1% Plasmocin prophylactic agent (Invivotech #ant-mpp) to obtain ten 150 mm culture dishes at 80% confluence. For irradiation, ten 150 mm culture dishes of 3T3 cells were trypsinized, centrifuged at 500 × g, resuspended in 30 mL 3T3 expansion culture medium and x-ray irradiated with a dose of 3000 cGy. The irradiated cells were collected by centrifugation and resuspended in 30 mL 3T3 cell freezing medium consisting of 90% FBS and 10% DMSO (Sigma-Aldrich #D2438) (3 ml freezing medium/150 mm dish of 3T3 cells) and stored in liquid nitrogen. When human feeder cells were needed, MRC-5 cells (ATCC #CCL-171) were expanded, irradiated, stored and used in a comparable manner.

### Airway epithelial cell expansion

One day prior to seeding plates with primary epithelial cells, irradiated 3T3 fibroblast feeder layers were plated on plasticware coated with Type IV Collagen (Sigma-Aldrich #C7521) in F + Y Reprogramming Medium (FYRM). FYRM consists of a mixture of DMEM 1.0 g/L glucose w/L-glutamine and w/ sodium pyruvate (Corning 10–014-CV) + Ham’s F-12 medium (Cytiva #SH30026.01; 1.8 g/L glucose) supplemented with 5% FBS, Insulin (5 µg/mL), Epidermal Growth Factor (10 ng/mL), Hydrocortisone (480 ng/mL), Adenine (24 µg/mL), Y-27632 (10 µM), Cholera Toxin (8.33 ng/mL), and antibiotics (Table 1), which was stored in foil-wrapped glass bottles in the dark at 4 °C for up to four weeks. One vial of 3T3 feeder cells was used for one T75 flask, three T25 flasks, or divided evenly in a 6-well tissue culture dish. Epithelial cell plating density is a key parameter in cell expansion; epithelial cells should be seeded at 1.3 × 10^5^ cells/well of a 6 well plate, 3.3 × 10^5^ cells/T25 flask, or 10^6^ cells/T75 flask. FYRM is changed every other day until the cells reach ~ 70% confluence. To remove epithelial cells cultured on 3T3 feeder layers, the 3T3 cells were first detached by washing with PBS/EDTA followed by a 5 min incubation in EDTA/PBS at RT, then light tapping. Rosettes of epithelial cells were detached using Accutase (Sigma-Aldrich #A6964) at room temperature for a maximum of 10 min and then reseeded for further expansion (one well to a T25 flask, one T25 to one T75 flask, or one T75 to three T75 flasks), plated for differentiation, or cryopreserved.

### Airway epithelial cell differentiation

E-ALI is based on a 50:50 mixture of DMEM containing 100 mg/dL glucose, w/o L-glutamine and w/sodium pyruvate (Sigma-Aldrich #D5546) and LHC Basal Medium (ThermoFisher #12,677–019) containing the additives summarized in Table 2. This recipe results in medium containing a final glucose concentration of 150 mg/dL. In each case, stock solutions are added to the medium prior to filtering (typically 1 ml of 1000 × stock/L medium). Most biologics were dissolved and aliquoted according to manufacturer’s instructions as 1000 × stock solutions stored frozen at -80 °C. Individual 1000 × stock solutions included 1000 × CaCl_2_·2H_2_O (1 M in H_2_O), 1000 × ZnSO_4_·7H_2_O (2 mM in H_2_O), 1000 × Fe/Mg (1.51 mM FeSO_4_·7H_2_O, 300 mM MgCl_2_·6H_2_O, 195 mM MgSO_4_·7H_2_O, 60 mM HCl in H_2_O). To make the 1000 × trace element stocks, first 1,000,000 × stocks were made in H_2_O for each component individually: Na_2_SeO_3_ (30 mM); MnCl_2_·4H_2_O (1 mM); Na_2_SiO_3_·9H_2_O (500 mM); (NH_4_)_6_Mo_7_O_24_·4H_2_O 1 mM; NH_4_VO_3_ (5 mM); NiSO_4_·6H_2_O (1 mM) and SnCl_2_·2H_2_O (0.5 mM). These individual stocks were diluted 1:1000 in H_2_O and HCl was added to 12 µM to produce the 1000 × trace elements stock. For anti-infectives, 1000 × stocks were made as follows, A: 154 mg Ceftazidime was added to 2 mL Gentamycin sulfate solution. B: 50 mg Cilistatin/Imipenem was added to 2 mL H_2_O. C: 88 mg Piperacillin and 12 mg Tazobactam were added to 2 mL DMSO. D: 100 mg Azithromycin and 10 mg Voriconazole were added to 2 mL DMSO. E-ALI is stored in foil-wrapped glass bottles in the dark at 4 °C for up to four weeks. Given the light sensitivity of E-ALI, medium changes are done in a biological safety cabinet with the fluorescent light turned off.

To produce differentiated cultures, P0 or expanded epithelial cells were resuspended in 20 mL E-ALI medium and counted. Cells were plated into 0.50 mL of E-ALI onto Type IV collagen coated Transwells at a density of 10^5^ cells/6.5 mm well (Costar #3470) or 3.5 × 10^5^/12 mm well (Costar #3460), with the bottom chamber containing 0.75 ml E-ALI. After 48 h, the basolateral medium was replaced with fresh E-ALI and the apical medium was removed to bring the cells to ALI. Once at ALI, medium was changed every 2–3 days, where the apical surface was washed once with E-ALI that was immediately removed and the basolateral medium was replaced. Benchmarks for differentiation included formation of a high resistance monolayer (> 500 Ohm x cm^2^) and initiation of cilia growth (day 7). Monolayers were usually fully differentiated 14–21 days after transition to ALI.

For some experiments, 2% Ultroser G medium containing normal resting glucose was made using 50:50 mixture of DMEM containing 100 mg/dL glucose, w/o L-glutamine and w/ sodium pyruvate (Sigma-Aldrich #D5546) and Ham’s F-12 medium (Hyclone #SH30026.FS; 180 mg/dL glucose).

### Cell stock cryopreservation and use

Epithelial cells were cryopreserved by diluting them into epithelial cell freezing medium (60% FYRM, 30% FBS, and 10% DMSO) at 1 million cells/mL using a Corning CoolCell according to the manufacturer’s directions. Frozen cell stocks were then transferred to liquid nitrogen for long-term storage. For cell thawing, each frozen vial was separately removed and immediately placed in a 37 °C water bath then left undisturbed until a small sliver of ice remained in the vial. The vials were then removed from the water bath, cleaned with 70% ethanol solution, and transferred to a biosafety cabinet for handling. Each vial was plated into either a single well of a 6-well dish or a T25, either of which was coated with Type IV collagen and containing 3T3 feeder cells, fully supplemented with room-temperature FYRM, avoiding centrifugation.

### Nasal epithelial cell isolation and expansion

Twenty-four hours prior to nasal cell collection, irradiated 3T3 fibroblast feeder cells were plated into 6-well cell culture dishes in Collection Medium consisting of DMEM containing 100 mg/dL glucose, w/o L-glutamine and w/ sodium pyruvate (Sigma-Aldrich #D5546) supplemented with 10% FBS (R&D Systems #S11150), 0.2% Primocin, and 0.1% Plasmocin treatment agent (Invivotech #ant-mpt), stored at 4 °C for up to two weeks. Nasal cell curettage was performed by a trained otorhinolaryngologist. A curettage was used to gently scrape each inferior nasal turbinate on both sides of the nose. Two separate scrapings were performed for each nostril to increase the number of isolated epithelial cells. Each curettage was placed into a 15 mL conical tube containing 3 mL Collection Medium. Nasal scrapes were then transported on ice for processing. The cells were dislodged from the curette by brief vortex. Nasal curettage samples from a single donor were then combined, centrifuged at 350 g for 5 min at RT, resuspended in PBS/EDTA, dissociated for 5 min at RT prior to straining through a 100 μM filter mesh and then centrifuged at 350 g for 10 min at RT. The dissociated cells were then resuspended in FYRM and placed onto irradiated 3T3 feeder cells at a density of one combined donor sample per well of a six-well culture plate that was precoated with Type IV collagen. After the initial two days of culture, the medium was changed with fresh FYRM daily until 60% confluence was reached. The cells were isolated as described above and then re-seeded in a T25 tissue culture flask containing irradiated 3T3 feeder cells in FYRM. At each passage, a portion of the cells were frozen in epithelial cell freezing medium (60% FYRM, 30% FBS, 10% DMSO) as illustrated in Fig. 5. Nasal epithelial cell differentiation was done using E-ALI as described above.

### Immunofluorescence and imaging

Antibodies used for immunofluorescence included: mouse monoclonal antibody (mAb) anti-acetylated tubulin at 1:100 (Sigma-Aldrich clone 6-11-B1; #T7451); mouse monoclonal antibody anti-Mucin 5AC at 1:500 (Abcam clone 2-11M1; #ab24071); rabbit polyclonal antibody anti-ZO-1 at 1:250 (ThermoFisher; #40-2300); mouse anti ZO-1 at 1:100 (ThermoFisher #33-9100), rabbit monoclonal antibody anti-Cytokeratin 5 at 1:250 (Abcam clone EP1601Y; #ab52635); rat monoclonal anti-Uteroglobin/SCGB1A1 at 1:50 (R&D Systems; MAB4218) and mouse monoclonal anti-FOXI1 clone OTI1D4 at 1:100 (Origene; TA800144). Cells were fixed in 4% paraformaldehyde in Dulbecco’s PBS containing Ca^2+^ Mg^2+^ (DPBS) for 10 min at RT, washed three times with DPBS, incubated with 1:1 MeOH:acetone for 2 min at RT, washed three times with DPBS, washed once with DPBS containing 0.5% Triton-X 100, blocked with DPBS  supplemented with 2% (wt/vol) BSA and 5% (wt/vol) goat serum for 1 h at RT, and then incubated with primary cell phenotype marker antibodies overnight in DPBS containing 2% BSA and 5% goat serum at 4 °C with mixing. The next day, cells were incubated with primary ZO1 antibody for 1 h at RT. Fluorescent secondary antibodies used were Cy2 Goat anti-mouse AffiniPure IgG (1:500; Jackson Immuno #115–165-166), Cy3 goat anti-rabbit AffiniPure IgG (1:500; Jackson Immuno #111–225-144), AlexaFluor568 goat anti-mouse IgG (1:1500; Invitrogen A-11031), AlexaFluor488 goat anti-rabbit IgG (1:1500; Invitrogen A-11034), or AlexaFluor488 donkey anti-rat IgG (1:500; Jackson Immuno #712–545-150). Secondary antibodies were diluted in DPBS supplemented with 2% BSA and 5% goat serum and incubated with cells for 1 h at RT. Cells were mounted in ProLong Gold Antifade Reagent with DAPI (Invitrogen; #P36931). Images were collected using a Zeiss FV1000 confocal microscope in the Emory University Integrated Cellular Imaging Microscopy Core.

### Transepithelial resistance and electrophysiology

To measure confluence and tight junction formation, transepithelial resistance (TER) was measured using an EVOM voltmeter, as previously described^61^. CFTR currents of cells on Transwells in physiologic Krebs-Ringers HEPES (KRH) buffer (1 g/l D-glucose, 50 mM HEPES, 137 mM NaCl, 4.7 mM KCl, 1.85 mM CaCl_2_, and 1.3 mM MgSO_4_ at pH 7.4) were measured as previously described^61^ with an Ussing chamber system (VCC MC-8 Multichannel Voltage/Current Clamp controller and analyzed using Acquire & Analyze software (Physiological Instruments)). Ion channel inhibitors and activators used for Ussing chamber analysis included amiloride (100 µM), forskolin (5 µM), 3-isobutyl-1-methylxanthine (IBMX; 100 µM), Vx-770 (5 µM), Vx-809 (3 µM), and CFTR inh172 (10 µM) and curcumin (40 µM).

### Insulin stimulation and glucose uptake

Medium glucose was measured using a colorimetric glucose quantification kit (Cayman Chemical; #10009582) and medium insulin was measured using by ELISA (Alpco Diagnostics; #80-INSHU-CH01). Uptake of 2-deoxy-D-[^3^H] glucose was measured as previously described^20^, with modifications. In brief, cells on Transwell permeable supports were washed with KRH and then incubated for 90 min at 37 °C in KRH. The cells were washed and incubated for 30 min at 37 °C with apically added KRH containing 0.5 μCi 2-deoxy-D-[^3^H]glucose (NEN Radiochemicals, PerkinElmer #NET328250UC) and 2-deoxy-D-glucose (Sigma-Aldrich #D6134) adjusted to 5.6 mM total final concentration in either the presence or absence of 500 nM (2.9 µg/ml) recombinant human insulin with zinc (Gibco #12585014). The cells were washed with cold KRH, Transwell filters were removed and placed in a scintillation vial containing 200 µL 0.1 M NaOH to lyse the cells. Scintillation fluid was added and the samples were measured for ^3^H using a Beckman-Coulter LS6500 scintillation counter.

### Statistics

All statistics were calculated using GraphPad Prism v6 for Windows with methods indicated in each figure legend. Cell doubling rate was calculated as (log(total cells)-log(number seeded cells)/log(2))/(time in culture).
